# Cysteine Transport through Excitatory Amino Acid Transporter 3 (EAAT3)

**DOI:** 10.1371/journal.pone.0109245

**Published:** 2014-10-02

**Authors:** Spencer D. Watts, Delany Torres-Salazar, Christopher B. Divito, Susan G. Amara

**Affiliations:** 1 Center for Neuroscience, Department of Neurobiology, University of Pittsburgh, Pittsburgh, Pennsylvania, United States of America; 2 Laboratory of Cellular and Molecular Neurobiology, National Institute of Mental Health, National Institutes of Health, Bethesda, Maryland, United States of America; Rosalind Franklin University, United States of America

## Abstract

Excitatory amino acid transporters (EAATs) limit glutamatergic signaling and maintain extracellular glutamate concentrations below neurotoxic levels. Of the five known EAAT isoforms (EAATs 1–5), only the neuronal isoform, EAAT3 (EAAC1), can efficiently transport the uncharged amino acid L-cysteine. EAAT3-mediated cysteine transport has been proposed to be a primary mechanism used by neurons to obtain cysteine for the synthesis of glutathione, a key molecule in preventing oxidative stress and neuronal toxicity. The molecular mechanisms underlying the selective transport of cysteine by EAAT3 have not been elucidated. Here we propose that the transport of cysteine through EAAT3 requires formation of the thiolate form of cysteine in the binding site. Using *Xenopus* oocytes and HEK293 cells expressing EAAT2 and EAAT3, we assessed the transport kinetics of different substrates and measured transporter-associated currents electrophysiologically. Our results show that L-selenocysteine, a cysteine analog that forms a negatively-charged selenolate ion at physiological pH, is efficiently transported by EAATs 1–3 and has a much higher apparent affinity for transport when compared to cysteine. Using a membrane tethered GFP variant to monitor intracellular pH changes associated with transport activity, we observed that transport of either L-glutamate or L-selenocysteine by EAAT3 decreased intracellular pH, whereas transport of cysteine resulted in cytoplasmic alkalinization. No change in pH was observed when cysteine was applied to cells expressing EAAT2, which displays negligible transport of cysteine. Under conditions that favor release of intracellular substrates through EAAT3 we observed release of labeled intracellular glutamate but did not detect cysteine release. Our results support a model whereby cysteine transport through EAAT3 is facilitated through cysteine de-protonation and that once inside, the thiolate is rapidly re-protonated. Moreover, these findings suggest that cysteine transport is predominantly unidirectional and that reverse transport does not contribute to depletion of intracellular cysteine pools.

## Introduction

Glutamate is the major excitatory neurotransmitter in the mammalian central nervous system (CNS). Following neurotransmitter release during synaptic transmission, glutamate is cleared perisynaptically by members of the excitatory amino acid transporter (EAAT) family. The EAAT family is composed of five members (EAAT 1–5), with EAAT1 and EAAT2 expressed primarily in glia, while EAAT3, EAAT4 and EAAT5 are mainly expressed in neurons of the CNS [1]–[3]. EAAT dysfunction results in elevated levels of glutamate, which have been associated with several neurological conditions such as ischemia, amyotrophic lateral sclerosis, Alzheimer’s disease, and epilepsy [1], [2], [4], [5].

Glutamate uptake proceeds by a secondary active transport mechanism which has been modeled as a multi-step cycle [6], [7]. The process is initiated by binding of co-transported ions (3 Na^+^, 1 H^+^) and substrate to the outwardly-oriented carrier, followed by translocation and release into the cytoplasm. Binding of an intracellular K^+^ ion drives the reorientation of the substrate binding site to an outward-facing conformation [7], [8]. Glutamate transport by EAATs has been shown to result in intracellular acidification associated with proton cotransport [7], [9]–[11]. Uptake of substrates by EAATs has also been shown to facilitate release of internal substrates [12]–[16], with substrate being translocated into the cell and exchanged for internal substrates that are then carried out of the cell as a result of reversibility of the translocation part of the transport cycle [15], [16].

In addition to L-glutamate, other acidic molecules such as L- and D-aspartate, cysteic acid, and serine-O-sulfate have been found to be substrates for the EAATs, while neutral amino acids such as serine and alanine have very low affinity (>1 mM) for the transporters [3], [17]. The specificity for high affinity binding and transport of acidic amino acids by EAATs involves a positively charged arginine residue, R447 in EAAT3, which is conserved across all EAATs [17]. In contrast, the neutral amino acid transporters (ASCT1 and ASCT2), which share sequence homology with the EAATs, transport the neutral amino acids serine, alanine and cysteine, and have the neutral residues threonine or cysteine respectively in the corresponding position [18], [19]. Substitution of R447 by cysteine in EAAT3 converts the protein from an acidic amino acid transporter to one that transports neutral amino acids [17].

**Figure 1:**
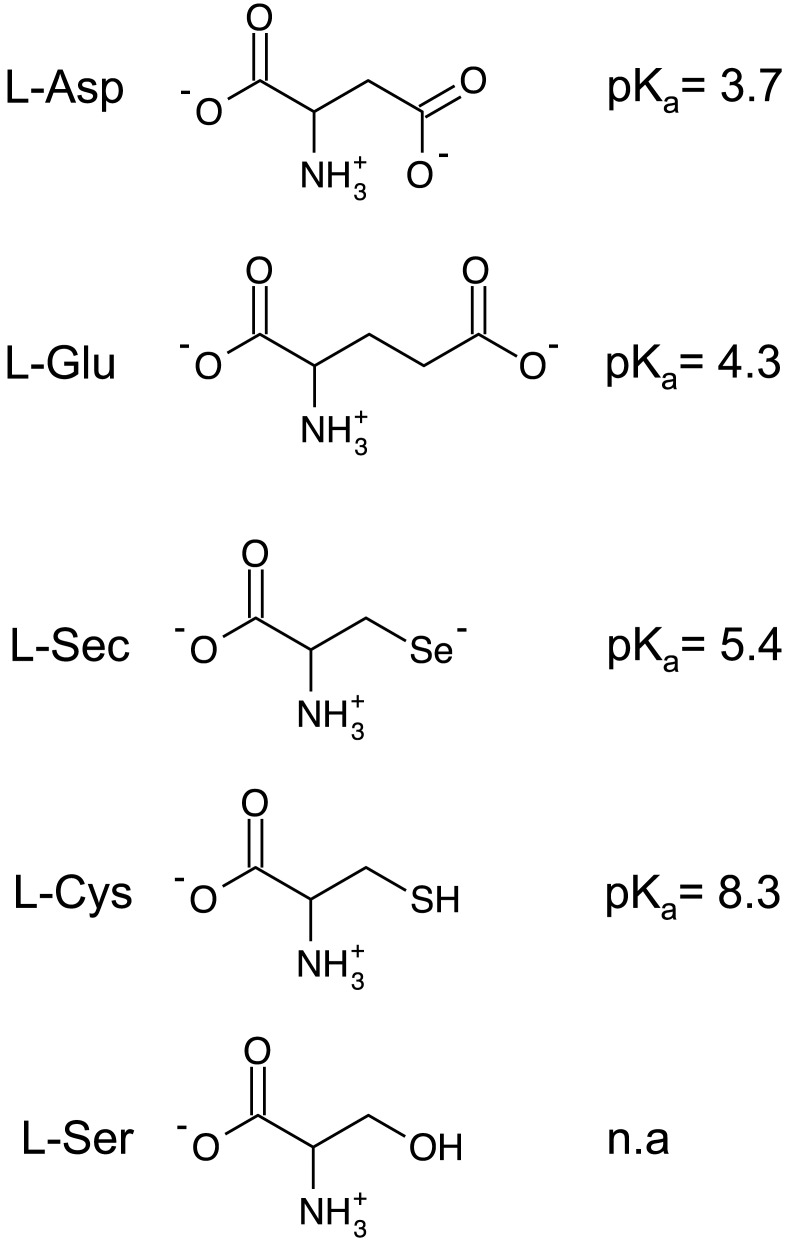
Structures of EAAT substrates and non-substrates. Diagrams of relevant amino acids and the associated side chain pK_a_s. Amino acids are depicted in their primary charge state at physiological pH. Abbreviations are: L-Asp, L-aspartate; L-Glu, L-glutamate; L-Sec, L-selenocysteine; L-Cys, L-cysteine; L-Ser, L-serine.

Selenium is an essential nutrient required in trace amounts and estimated to be specifically incorporated as selenocysteine in more than 20 human proteins. Many of these proteins use selenocysteine as an active site residue and are critical for maintenance of cellular redox potential and repair of oxidative damage [20]–[22]. Selenocysteine is a primary source of selenium for the selenophosphate required for tRNA^Sec^ synthesis [23]. Selenocysteine is structurally similar to cysteine (Figure 1) with substitution of selenium for the sulfur of cysteine. A primary effect of this substitution is a lower pK_a_ (5.3) for selenocysteine, resulting in a deprotonated and negatively charged side chain at physiological pH, similar to glutamate, whereas cysteine (pK_a_ = 8.4) is primarily protonated. While it is clear that selenocysteine uptake into cells occurs, no transport system has been identified.

EAAT3, which is selectively expressed on neurons in the CNS, also transports L-cysteine with an approximately 10-fold higher apparent affinity for transport (K_m_) and a much larger transport rate than the other members of the family [13]. Maintaining sufficient intracellular concentrations of cysteine is vital not only for protein synthesis but also for maintenance of cellular redox homeostasis as cysteine is the rate limiting component for the synthesis of glutathione, a critical co-factor of the intracellular antioxidant machinery. Cysteine transport by EAAT3 has been implicated in playing a significant role in maintenance of the intracellular redox potential [4]. Indeed, in EAAT3 deficient mice, the dopaminergic neurons of the substantia nigra pars compacta, a neuronal population highly susceptible to oxidative stress, show decreased levels of neuronal glutathione, increased evidence for oxidative stress and decreased neuronal survival during aging [24]. Treatment of these mice with the cysteine analogue N-acetylcysteine (NAC), which is taken up by cells in an EAAT3 independent manner, rescues the phenotype supporting the idea that EAAT3 activity is important for cellular cysteine homeostasis [4], [25].

Here we examined cysteine transport through EAAT3. Based on the structural similarities of selenocysteine with cysteine and charge similarities between selenocysteine and glutamate, we hypothesized that selenocysteine uptake could occur through transport by the EAATs and provide a useful tool for understanding cysteine transport by EAATs. We demonstrate that selenocysteine can be readily transported by the human glutamate transporter isoforms, EAAT1, EAAT2 and EAAT3, and validate that these carriers transport selenocysteine with apparent affinities and transport capacities comparable to glutamate. By comparing the transport characteristics of selenocysteine with cysteine and monitoring transport using a pH-biosensor, our results suggest a mechanism in which EAAT3 facilitates cysteine transport through cysteine de-protonation. Moreover, our results indicate that the mechanism favors inwardly-directed transport of cysteine and does not contribute to the depletion of intracellular cysteine pools.

## Materials and Methods

### mEGFPpH Construct Preparation

mEGFPpH was constructed in our laboratory from EGFP using site-directed mutagenesis and addition of an N-terminal palmitoylation sequence as follows. First, EGFP-H148D [26] (EGFPpH) was generated in the mammalian expression plasmid pEGFP-N3 (Clontech) by a single nucleotide substitution using site directed mutagenesis with the following primers: (sense) CTGGAGTACAACTACAACAGC**G** ACAACGTCTATATCATGGCCGAC and (antisense) GTCGGCCATGATATAGACGTTGT**C**GCTGTTGTAGTTGTACTCCAG [C -> G base change (bold) to create the CAC -> GAC codon change (underlined)]. Mutagenesis was verified by DNA sequencing of the EGFPpH reading frame in plasmid pEGFPpH. Addition of the palmitoylation sequence to EGFPpH to provide advantages of membrane targeting [27] was accomplished as follows. Plasmid pEGFPpH was digested at the KpnI and BamHI sites immediately 5′ of the ATG start codon for EGFPpH. Two complementary oligos (sense: cGCCGCCACCATGCTGTGCTGTATGAGAAGAACCAAACAGGTTGAAAAGAATGATGAGGACCAAAAGATCg; antisense: gatccGATCTTTTGGTCCTCATCATTCTTTTCAACCTGTTTGGTTCTTCTCATACAGCACAGCATCAGCATGGTGGCGGCggtac; small letters denote restriction site nucleotides) including Kozak site (GCCACCACCATGC) [28] and coding for the palmitoylation signal peptide from neuromodulin (GAP-43), MLCCMRRTKQVEKNDEDQKI [27], [29] were 5′-phosphorylated then hybridized to form a double stranded insert and subsequently ligated into the cut plasmid. Transformants were screened by colony PCR for insertion of the palmitoylation sequence with the following primers: forward, ATGCTGTGCTGTATGAGAAGA (palmitoylation tag); reverse, TTACTTGTACAGCTCGTCCAT (3′-EGFP sequence). Positive colonies were subjected to DNA sequencing of the entire mEGFPpH reading frame to verify proper insertion and H148D mutation. A single positive transformant was expanded for DNA preparation (GenElute HP, Sigma-Aldrich) for HEK293 cell transfection and protein expression.

### Cell Culture and Protein Expression

HEK293 cells were cultured at 37°C in a humidified 5% CO_2_ incubator in DMEM (Lifetech, Gibco BRL, Invitrogen) supplemented with 10% FBS, penicillin (100 U/mL) and streptomycin (100 µg/mL). For characterization of the pH response of mEGFPpH, 1×10^5^ cells/mL were batch transfected in 1 mL of Opti-Mem (LifeTechnologies) using 6 µL Lipofectamine 2000 (Invitrogen) and 1 µg pmEGFPpH with 2×10^4^ cells plated on poly-L-Lysine coated 15 mm coverslips. Cells were incubated as above for two days prior to NH_4_Cl experiments.

For imaging experiments monitoring the intracellular acidification associated with glutamate transport, 1×10^5^ cells/mL were batch transfected in 1 mL of Opti-Mem (LifeTechnologies) using 6 µL Lipofectamine 2000 (Invitrogen) and 0.5 µg pCMV-EAAT3 [3] and 0.5 µg pmEGFPpH) with 2×10^4^ cells plated on poly-L-Lysine coated 15 mm coverslips in 12 well plates with 1 mL DMEM culture media as above. Cells were incubated at 37°C in a humidified 5% CO_2_ for two days prior to imaging experiments.

For radiolabeled transport experiments, 2×10^6^ cells were transfected in 1 mL of Opti-Mem (Life Technologies) using 6 µL of Lipofectamine 2000 (Invitrogen) with 1 µg pCMV-EAAT2 or pCMV-EAAT3 DNA and plated at 5×10^4^ cells/well in 24 well Costar (Corning) plates with 0.5 mL DMEM culture media prepared as above. Following transfection and plating, cells were cultured 2–3 days prior to [^3^H]-L-Glutamate transport experiments.

### mEGFPpH Fluorescence Imaging in HEK293 cells

Cells expressing mEGFPpH were imaged with a Zeiss Axiovert 135T inverted microscope with a 40X NeoFluar oil immersion objective (NA1.3). A Lambda DG4 (Sutter Instrument Co.) equipped with a xenon arc lamp was used for high-speed excitation wavelength switching. Images were collected using either a Quantix (Roper Scientific, Tucson, AZ) or an Orca ER (Hammamatsu Co.) cooled CCD. For pH imaging experiments, the fluorescence filters (Omega Optical) were: excitation 405±20 and 485±7.5; dichroic 400-480-585DLRP; emission 510±11.5. Software control and data acquisition were accomplished using Axon Imaging Workbench (INDEC Biosystems) or OpenLab acquisition software (Improvision, Perkin Elmer). Cells were incubated at room temperature with continuous perfusion using standard buffer containing (in mM): 146 NaCl, 5 KCl, 5 HEPES, 2.5 CaCl2 and 1.2 MgCl2 at pH 7.35 with additions (substrates etc.) as indicated in figure legends. Acquisition exposure times were identical for both 405 nm and 485 nm excitation wavelengths adjusted so that the 510 emission was not saturated for either excitation wavelength. Prior to perfusion with buffers containing substrates, cells were perfused in standard buffer to achieve a stable base line. Fluorescence was reported as the ratio of fluorescence at 510 nm by excitation with 485 and 405 nm wavelengths (F485/F405).

For NH_4_Cl experiments, HEK293 cells expressing mEGFPpH were perfused with buffer to establish a stable fluorescence baseline, followed by perfusion with buffer containing 50 mM NH_4_Cl substituted for 50 mM NaCl, followed by NH_4_Cl washout with standard buffer as indicated. Calibration of fluorescence ratio changes with intracellular pH was accomplished following the NH_4_Cl experiments, by perfusion of the cells with standard buffer containing 10 µM each of nigericin and valinomycin at the following pH: 6.5, 7.35, 7.5, 7.75, 8, 8.5. Perfusion at each pH was continued till a stable fluorescence ratio was achieved, upon which perfusion with the next higher pH buffer was begun.

### Expression of Constructs in *Xenopus* oocytes

mRNA from linearized pOTV-EAAT1, pOTV-EAAT2 and pOTV-EAAT3 was generated as previously described [30] and resuspended in RNAase-free water and used for oocytes injections. *Xenopus oocytes* where injected with 50 nl of mRNA and incubated at 18°C in 96 mM NaCl, 2 mM KCl, 1.8 mM CaCl_2_, 1.0 mM MgCl_2_, 5 mM HEPES, pH 7.4 (ND96) supplemented with 2.5 mM Sodium Pyruvate and Gentamicyn for 2 to 3 days prior to using in electrophysiological recordings and radio-labeled transport experiments.

### Electrophysiological Recording in *Xenopus* oocytes

Glutamate transporter-associated currents were recorded by two-electrode voltage clamp using a GeneClamp 500B (Molecular Devices, Sunnyvale, CA, USA). Oocytes were held at −60 mV, and currents elicited by 200 ms voltage steps between −120 mV and +60 mV were filtered at 2 kHz (−3dB) and digitalized with a sampling rate of 10 kHz using a Digidata AD/DA converter (Molecular Devices, Sunnyvale, CA, USA). Borosilicate pipets where filled with 3 M KCl and typical resistances where 0.2 to 1 MΩ. Transport currents were determined at −60 mV in a gluconate-based ND96 solution. In order to dialyze internal chloride, oocytes were incubated 12 to 24 hours in the gluconate solution. Anion currents were determined at positive potentials (+60 mV) after exchanging the external solution to NO_3_-based ND96 (replacement of 96 mM NaCl with 96 mM NaNO_3_) solution in the absence or in the presence of 1 mM external substrates. In experiments using cysteine as a substrate, all solutions contained 2 mM dithiothreitol (DTT). Data were analyzed with a combination of pClamp 9.2 (Axon Instrument) and Sigma Plot (Jandel Scientific, San Rafael, CA, USA). Whole cell current amplitudes are shown without any subtraction protocol. Current-voltage relationships were constructed by plotting the isochronal current 6 ms after the voltage step versus the membrane potential. All data are given as the mean ± standard deviation of the mean (SDM). For evaluation the statistical relevance, the Student’s t-Test was used with an α of 0.05.

### Substrate inhibition of [^3^H]-L-glutamate uptake in HEK293 cells

For cysteine and selenocysteine inhibition of glutamate transport, HEK293 cells expressing EAAT3 or EAAT2 were incubated in perfusion buffer with 20 nM [^3^H]-L-glutamate with non-radiolabeled L-glutamate added for a final concentration of 30 µM glutamate in the presence of varying concentrations of cysteine or selenocysteine as indicated. Transport was allowed to proceed for 10 min and was terminated by 3 washes with cold perfusion buffer. Cells were lysed with 1% SDS/0.1N NaOH and radioactivity determined by scintillation counting. Data analysis was performed using Graphpad Prism (Graphpad Software Inc.).

### Cysteine transport and glutamate inhibition of cysteine transport in *Xenopus* oocytes

Cysteine transport was measured for 10 min in *Xenopus* oocytes with serial dilution of a mixture of 10 mM cysteine and 20 nM [^35^S]-L-cysteine in ND96. Transport was terminated by three washes of ND96, after which accumulated radiolabel was quantitated by liquid scintillation counting. For glutamate inhibition of cysteine transport, oocytes were incubated with a fixed concentration of [^35^S]-L-cysteine and increasing concentrations of glutamate in ND96 at pH 6.9 or pH 8.5. Transport was terminated by three washes in ND96 followed by liquid scintillation counting. The percentage of thiolate to thiol cysteine was calculated using the Henderson–Hasselbalch equation and a cysteine sulfhydryl (R-SH) pK_a_ of 8.3.

### Glutamate uptake and hetero-exchange assays in *Xenopus* oocytes

For experiments in *Xenopus oocytes,* oocytes co-expressing EAAT3 and ASCT1 were preincubated with either 20 nM [^35^S]-L-cysteine for a final concentration of 300 µM cysteine or 20 nM [^3^H]-L-glutamate for a final concentration of 30 µM glutamate incubated for 20 min. Oocytes were washed 3 times in ND96 buffer followed by incubation for 10 min with non-radiolabeled substrates as indicated. The supernatant was removed and collected for scintillation counting, while the oocytes were lysed in 1% sodium dodecylsulfate (SDS). The oocytes lysate and supernatant were counted separately by liquid scintillation counting to determine both retained and released radioactivity respectively. Percent of released radioactivity was determined as a function of the released/(retained + released).

## Results

### Selenocysteine induces transport currents in EAAT expressing oocytes

**Figure 2:**
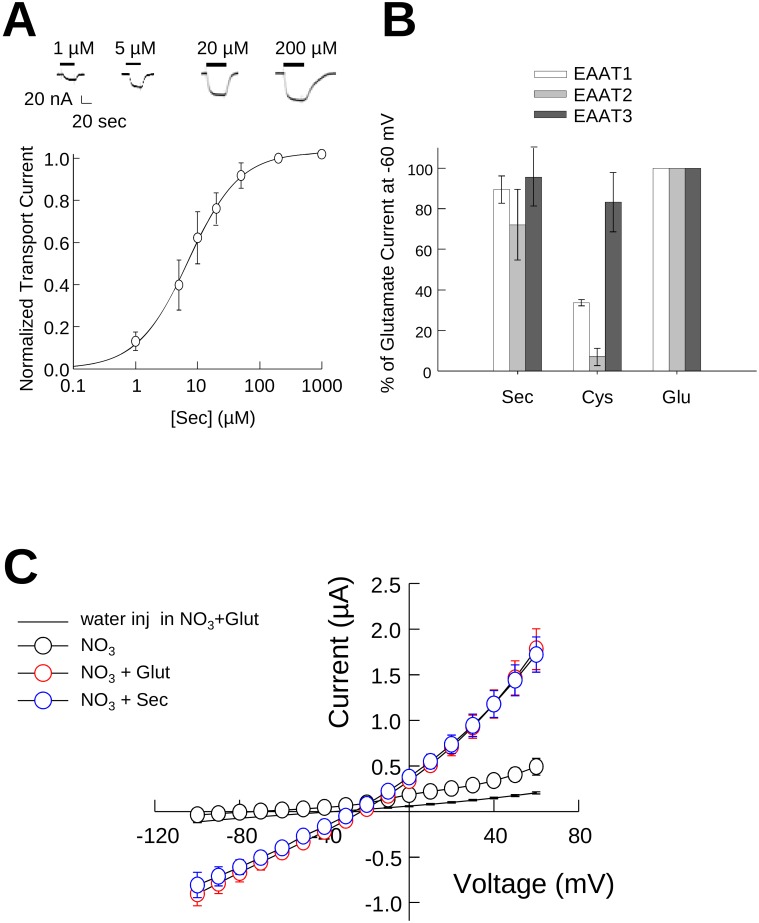
Selenocysteine is transported by EAATs 1–3. **A)** Representative recordings (upper panel) and averaged normalized transport currents (lower panel) measured at −60 mV as a function of the L-selenocysteine concentration in EAAT3 expressing oocytes (n = 6). Data are presented as the mean and Std. dev. of the mean and fit with the Hill equation to estimate the Km for transport. **B)** Comparison of the maximal transport currents at −60 mV for L-selenocysteine and L-cysteine by EAAT1 (n>3), EAAT2 (n>5) or EAAT3 (n>10) normalized to the maximal currents induced by L-glutamate measured in the same oocyte. **C)** Comparison of averaged current-voltage relationships recorded from oocytes expressing EAAT3 for both 1 mM glutamate (red symbols, n = 4) and 1 mM selenocysteine (blue symbols, n = 4). Black symbols indicate the averaged current voltage relationship of the same cells in the absence of substrate (n = 4) and the solid line represents the average of water injected oocytes in the presence of 1 mM glutamate (n = 5).

Selenocysteine is structurally similar to cysteine but at physiological pH the selenium side chain is deprotonated (pK_a_ = 5.3) and negatively charged similar to glutamate and aspartate (Figure 1). Based on these characteristics we predicted that selenocysteine would be a substrate for the glutamate transporters. To test this, we first measured selenocysteine transport currents using two-electrode voltage clamp in *Xenopus* oocytes expressing EAAT3. Perfusion of EAAT3 expressing oocytes with 1 µM selenocysteine induced an inward transport current of 20 nA (Figure 2A, top). Perfusion with increasing concentrations of selenocysteine resulted in a corresponding increase in the transport current amplitude. Normalizing the current amplitude to the maximal selenocysteine induced transport current and plotting as a function of the applied selenocysteine concentration (Figure 2A, bottom) exhibited a concentration dependence well described by the Michaelis-Menten equation with an apparent affinity of 7.14±0.3 µM (n = 6). This was not observed in uninjected oocytes (data not shown).

As a further characterization of selenocysteine transport, we measured the current obtained by perfusion of 1 mM selenocysteine and cysteine relative to the maximal current obtained upon application of a saturating 1 mM glutamate in *Xenopus* oocytes (Figure 2B). We found that selenocysteine induced currents in oocytes expressing EAATs were similar to those observed for glutamate, with current amplitudes of 89.5±6.7% (n = 5), 72.1±17% (n = 7) and 95.6±14% (n = 7) of the glutamate transport current for EAAT1, EAAT2 and EAAT3 respectively (Figure 2B). In contrast to selenocysteine, the currents measured by perfusion of 1 mM cysteine in oocytes expressing EAAT1 or EAAT2 were only 33±1.5% (n = 3) and 7.0±4.2% (n = 5) of that for 1 mM glutamate respectively, consistent with the reported poor cysteine transport properties of these carriers [13]. In comparison, cysteine induced a robust transport current in EAAT3 expressing oocytes, with a current amplitude that was 86±14% (n = 5) of that produced by 1 mM glutamate (Figure 2B). These results indicate that at physiological pH selenocysteine is a substrate not only for EAAT3, but for EAAT 1 and 2 as well.

EAAT3 has an uncoupled anion conductance which is activated by Na and glutamate [31], [32]. To further characterize the interaction of selenocysteine with EAAT3, using two electrode voltage-clamp we compared the current-voltage relationship for the anion current gated by 1 mM selenocysteine and 1 mM glutamate in EAAT3 expressing oocytes (Figure 2C). After perfusion with a NO_3_-based solution in the absence of substrate, at +60 mV we obtained a macroscopic current amplitude of 0.5±0.09 µA (black open circles, n = 4). Application of 1 mM glutamate to the same cells elicited a 3-fold larger current with an amplitude of 1.78±0.4 µA (red open circles, n = 4), as previously observed [33]. Upon application of 1 mM selenocysteine, we obtained a very similar current-voltage relationship with a current amplitude at +60 mV of 1.72±0.4 µA (blue open circles, n = 4) (Figure 2C). Current amplitudes from control oocytes in the presence of substrate were 0.2±0.001 µA (solid line, n = 5). These results demonstrate that selenocysteine activates the EAAT3-mediated anion conductance in a fashion similar to glutamate.

### Inhibition of glutamate transport by Selenocysteine and cysteine

**Figure 3:**
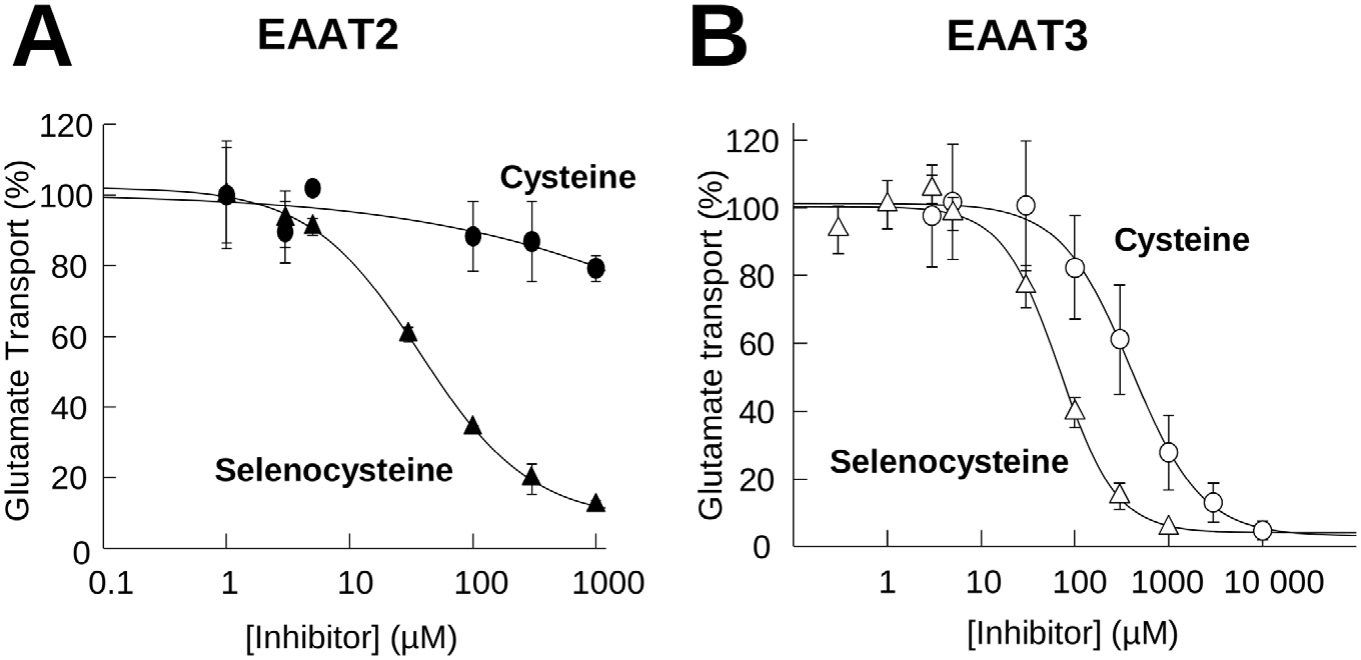
Inhibition of glutamate transport by L-Selenocysteine and L-Cysteine for EAAT 2 and EAAT 3. Inhibition of radiolabeled glutamate uptake using varying concentrations of L-cysteine (circles) or L-selenocysteine (triangles) in HEK293 cells expressing EAAT2 (**A,** n>5 for each data point) or EAAT3 (**B,** n>5 for each data point). Data are represented as the mean and the standard error of the mean with non-linear curve fit to calculate the IC_50_s.

Substrates for the EAATs can act as competitive inhibitors of glutamate uptake. Using a fixed concentration of radiolabeled glutamate and varying the substrate-inhibitor concentration, an IC_50_ can be calculated providing a measure of the relative binding affinity of the substrate for the transporter. Using this approach, we calculated the selenocysteine and cysteine IC_50_s for inhibition of radiolabeled glutamate uptake. Incubation of EAAT2 expressing HEK293 cells with varying concentrations of selenocysteine resulted in a dose dependent inhibition of glutamate transport, with ∼90% inhibition at 1 mM selenocysteine and a calculated IC_50_ of 46±12 µM (n = 3) (Figure 3A). The cysteine inhibition curve of glutamate transport in EAAT2 expressing cells was incomplete under these assay conditions, with only a 20% inhibition at the maximum concentration of 1 mM cysteine (Figure 3A).

For EAAT3 expressing cells, cysteine inhibition of glutamate uptake was substantially greater than that observed for EAAT2 with 70% inhibition at 1 mM cysteine and an IC_50_ of 631±50 µM (n = 3). The concentration dependence for selenocysteine inhibition of glutamate transport by EAAT3 expressing cells was shifted to the left when compared to that of cysteine with >90% inhibition at 1 mM selenocysteine and an IC_50_ of 71±10 µM (n = 3), approximately 10-fold lower than for cysteine (Figure 3B).

### Effect of pH on glutamate inhibition of cysteine transport

**Figure 4:**
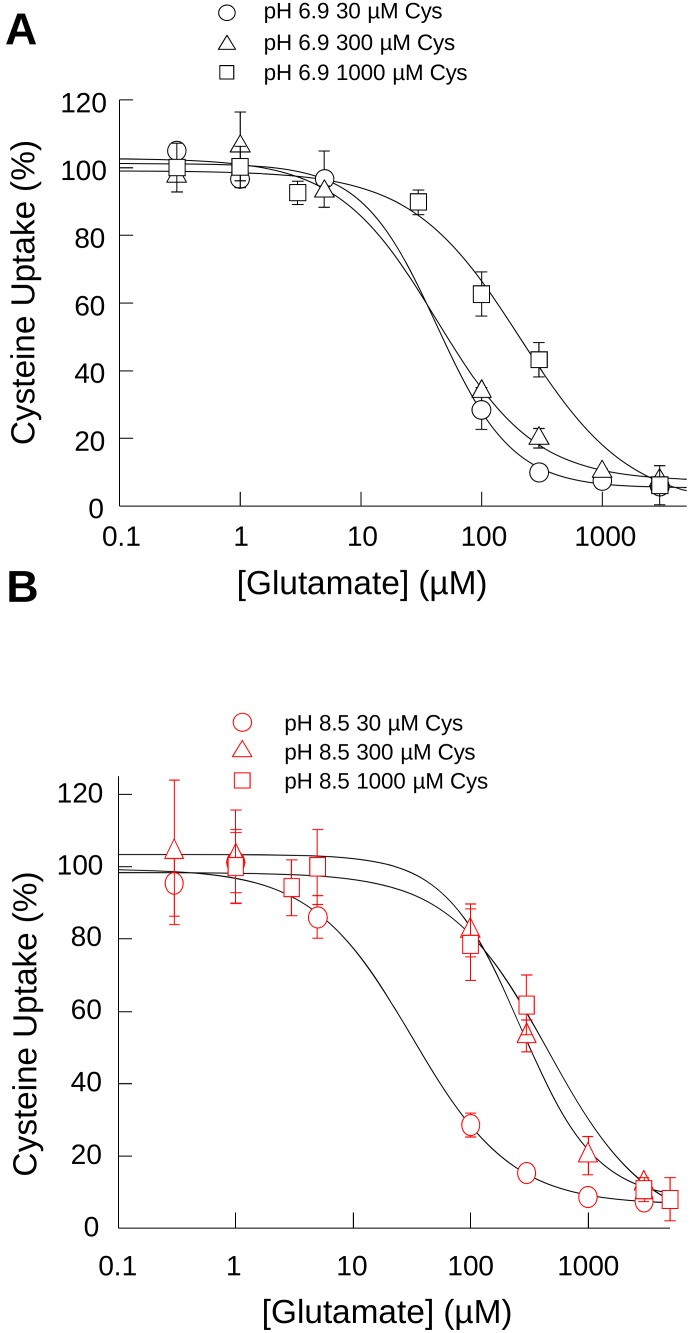
pH affects glutamate inhibition of cysteine transport. Glutamate inhibition of cysteine uptake from oocytes expressing EAAT3 at three different cysteine concentrations: 30 µM (circles), 300 µM (triangles) and 1 mM (squares) at pH 6.9 (**A**) and pH 8.5 (**B**).

Selenocysteine and cysteine differ in side chain charge at physiological pH with selenocysteine existing primarily in a deprotonated (>99%) state at physiological pH compared to cysteine which is predominantly protonated (∼90%). For cysteine, the thiolate (R-S^−^) form increases to ∼61% at pH 8.5. As glutamate has been reported to be a potent inhibitor of cysteine transport [34] at physiological pH, we hypothesized that increasing the thiolate to thiol ratio would alter the inhibitory properties of glutamate toward [^35^S]-cysteine uptake. To test this, we assayed [^35^S]-cysteine uptake at three cysteine concentrations for a range of glutamate concentrations in buffers of pH 6.9 (Figure 4A) and pH 8.5 (Figure 4B), where cysteine is ∼3.8% and 61% thiolate, respectively. At pH 6.9, the curves obtained for glutamate inhibition of 30 µM and 300 µM cysteine were similar, with ∼75% inhibition of cysteine uptake for both at 100 µM glutamate with similar IC_50_s for glutamate of 42.8±9.8 µM (n = 3) and 42.5±12.1 µM (n = 3) respectively. At the 1 mM cysteine concentration, the glutamate inhibition curve was shifted to the right, with 100 µM glutamate inhibiting cysteine transport by only 40% and an approximate 5 fold increase in the IC_50_ to 210.3±58.8 µM (n = 3) (Figure 4A).

When we performed the assay at pH 8.5, where the thiolate represents ∼61% of the total cysteine, there was minimal difference of the curve obtained for 30 µM cysteine compared to that obtained for the same concentration assayed at pH 6.9, with a calculated IC_50_ of 31.5±7.13 µM (n = 3) (Figure 4B). However, at 300 µM cysteine, the glutamate inhibition curve was shifted to the right with a calculated IC_50_ of 267.1±12.1 µM (n = 3), a five-fold increase compared to the same cysteine concentration assayed at pH 6.9. For the 1 mM cysteine concentration at pH 8.5, the glutamate inhibitory curve did not shift substantially compared to that obtained at pH 6.9, with a calculated IC_50_ of 455.5±127.8 µM (n = 3). These data demonstrate that EAATs preferentially interact and translocate the thiolate form of cysteine, a likely necessity for satisfying interactions with TM8 residue R447 [17].

### Glutamate transport activity monitored by pH

**Figure 5:**
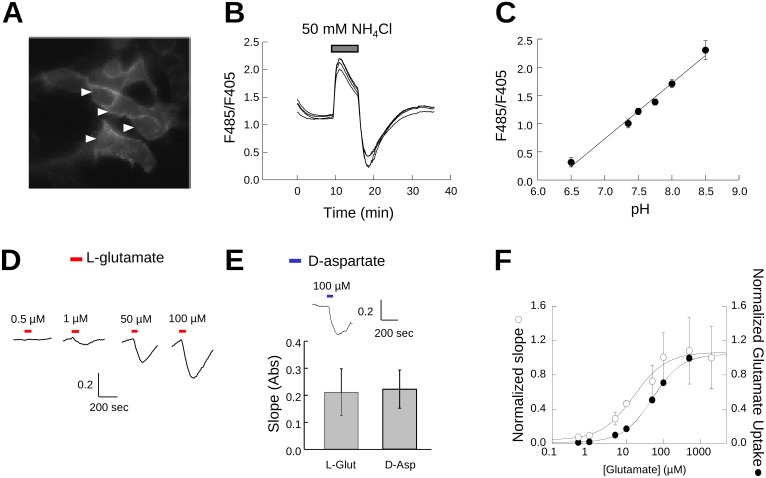
mEGFPpH detects intracellular pH changes induced by glutamate transport. Representative image of mEGFPpH transfected HEK293 cells (**A**) and representative fluorescence traces from HEK293 cells expressing mEGFPpH perfused with 50 mM NH_4_Cl (**B**). Y axis indicates the ratio of fluorescence emission at 510 nm from excitation at 485 nm and 405 nm (F485/F405) (**B**). Arrows in **A** indicate the cells from which the traces in **B** were recorded. **C)** Fluorescence ratio (F485/F405) as a function of induced intracellular pH following NH_4_Cl perfusion (**B**). **D)** Perfusion of increasing concentrations of L-glutamate results in increased rate of mEGFPpH fluorescence decrease in HEK293 cells co-transfected with EAAT3 and mEGFPpH. The Y-axis units are the fluorescence ratio for emission at 510 nm with excitation at 485 and 405 nm (F485/F405). **E)** Perfusion with 100 µM D-aspartate results in intracellular acidification with slope magnitude similar to that for 100 µM L-glutamate (bar graph). Y-axis units are the fluorescence ratio for emission at 510 nm with excitation at 485 and 405 nm (F485/F405). **F)** Representation of the magnitude of the slope of mEGFPpH fluorescence ratio decrease (left y-axis) as a function of the applied glutamate concentration compared with the glutamate transport activity (right y-axis) in similarly transfected cells.

As an additional method to compare selenocysteine and cysteine transport by EAATs, we made use of a fluorescence assay to monitor the intracellular acidification resulting from proton co-transport associated with the EAAT transport cycle [7], [9], [10]. This assay is based on a pH-biosensor derived from the enhanced green fluorescent protein (EGFP) with a single amino acid substitution (H148D) to detect pH changes resulting from the inward movement of protons by EAATs. The H148D amino acid substitution results in an EGFP-based biosensor (EGFPpH) with a fluorescence intensity directly proportional to pH over a physiologically relevant range [26]. An N-terminal palmitoylation signal sequence was added to anchor the pH sensor to the cytoplasmic face of cellular membranes (mEGFPpH) to position the fluorescence sensor proximal to the region of transporter mediated proton flux. In transfected HEK293 cells the fluorescence is largely localized at the plasma membrane, as evidenced by the increased fluorescence at the periphery of the cell (Figure 5A) indicative of membrane targeting expected following palmitoylation.

To test the mEGFPpH responsiveness to changes in intracellular pH, we used NH_4_Cl perfusion and washout to induce alkaline and acidic intracellular conditions respectively [35]. In HEK293 cells expressing mEGFPpH, perfusion with NH_4_Cl induced a rapid increase in mEGFPpH fluorescence which peaked and was followed by a steady decrease in mEGFPpH fluorescence (Figure 5B). NH_4_Cl washout induced a rapid decrease in mEGFPpH fluorescence which reached a minimum and was followed by a slow return to baseline. Calibration of mEGFPpH fluorescence as a function of pH shows the fluorescence response to be linear over the range of pH 6.5 to pH 8.5 (Figure 5C). In this experiment, we observed an average initial intracellular pH (pH_i_) of ∼7.4 (n = 4) prior to NH_4_Cl perfusion, with the NH_4_Cl perfusion increasing the average pH_i_ to ∼8.5 and washout dropping the pH_i_ to ∼6.5. This demonstrates that the pH sensitivity of membrane attached mEGFPpH is similar to that previously reported for the EGFP H148D mutation [26] and is suitable for detection of pH_i_ changes over a physiologically relevant range.

To test whether mEGFPpH was capable of detecting pH_i_ changes resulting from proton movement coupled to glutamate transport, mEGFPpH was co-expressed with EAAT3 in HEK293 cells and the fluorescence of mEGFPpH monitored during exposure of the cells to glutamate. Based on the results obtained during the NH_4_Cl experiments described above, intracellular acidification due to proton co-transport would be expected to decrease pH_i_, resulting in a decrease in mEGFPpH fluorescence. When cells were perfused with a relatively low concentration of glutamate (0.5 µM), no observable fluorescence changes from baseline were observed (Figure 5D). Increasing the perfused glutamate concentration to 1 µM resulted in a mEGFPpH fluorescence decrease during the glutamate pulse, with 50 µM and 100 µM glutamate resulting in more robust mEGFPpH fluorescence decreases (Figure 5D). To confirm that the fluorescence changes are the result of movement of H^+^ coupled to glutamate transport and not to H^+^ generated through increased metabolic processes due to increased intracellular glutamate, cells were perfused with the non-metabolized EAAT substrate D-aspartate. Perfusion with 100 µM D-aspartate induced a fluorescence decrease similar to that observed for 100 µM glutamate (Figure 5E), indicating that the fluorescence changes are coupled to EAAT transport activity and not metabolism. The fluorescence changes were blocked by co-application of glutamate with the EAAT transport inhibitor TBOA and were not readily observed in cells expressing mEGFPpH alone (data not shown). We observed that the slope or rate of the fluorescence decrease varied with glutamate concentration and therefore was reflective of glutamate transport rate suggesting that this method could be used to assay glutamate transport activity. A similar approach using fluorescent proteins to assay membrane transport has been described for CFTR using a GFP derived halide sensor [36]. To confirm that the slope of the fluorescence response varies with glutamate concentration, we measured the maximal steady state slope of the mEGFPpH fluorescence decrease resulting from glutamate transport activity for a range of glutamate concentrations from 0.5 µM to 2 mM. As we found that the magnitude of the slope varied from cell to cell, likely as a result of variation in the expression of EAAT3, we normalized the slope magnitude to that obtained for 1 mM glutamate in the same cell. Plotting the slope magnitude as a function of the applied glutamate concentration resulted in a dose response curve with a calculated Km of 30 µM (Figure 5F). This compares to a K_m_ of 70 µM calculated for radiolabeled glutamate uptake from similarly transfected cells. These results validate this approach of using a pH-biosensor to assay glutamate transporter activity through monitoring pH_i_.

### EAAT3 cysteine transport increases pH_i_ whereas selenocysteine decreases pH_i_

**Figure 6:**
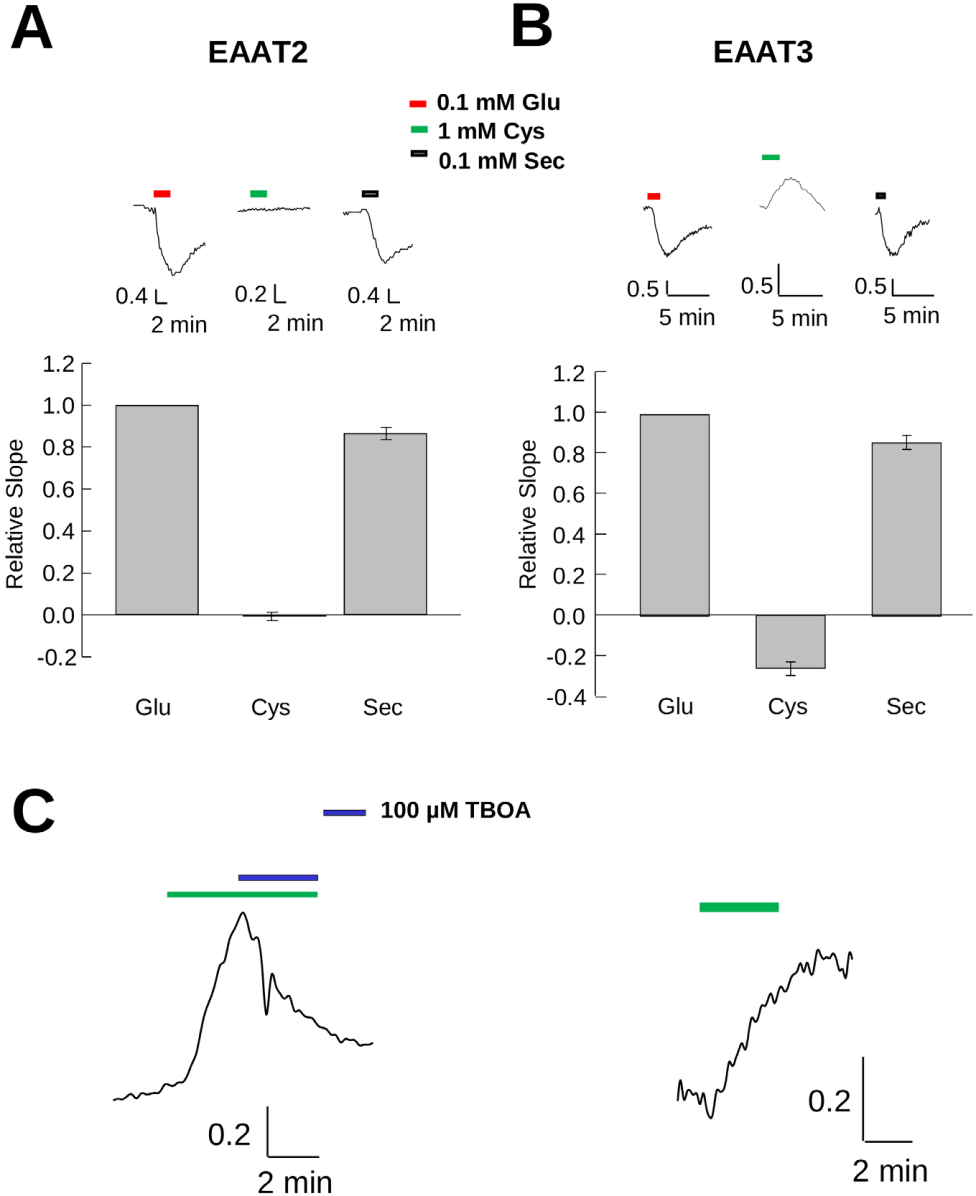
Transport of substrates in cells expressing EAAT2 or EAAT3 differentially affects intracellular pH. Representative fluorescence recordings from HEK293 cells expressing EAAT2 (**A**) or EAAT3 (**B**) in response to short applications of different concentrations of L-cysteine, L-glutamate or L-selenocysteine. The magnitude of maximal steady state slopes (**A** and **B**) are plotted in bar graphs below each trace, normalized to the glutamate slope magnitude. **C)** Representative trace of the effect on cysteine induced mEGFPpH fluorescence changes in EAAT3 expressing HEK293 cells with (left) or without (right) 100 µM TBOA. The Y-axis units for traces represent the fluorescence ratio for emission at 510 nm with excitation at 485 and 405 nm (F485/F405).

To better characterize the transport properties of cysteine and selenocysteine, we used mEGFPpH to monitor changes in pH_i_ associated with transport of either substrate and compared the results to those observed with glutamate. In HEK293 cells co-expressing mEGFPpH and EAAT2, a carrier which has low affinity (K_m_>1 mM) [13] and a very low capacity for cysteine transport (Figure 2B), perfusion of 1 mM cysteine (Figure 6A) did not produce observable fluorescence changes from baseline. However, perfusion of 1 mM glutamate induced a significant quench of mEGFPpH fluorescence in the same cells (Figure 6A), consistent with a pH_i_ decrease due to coupled proton co-transport. Perfusion of these cells with 1 mM selenocysteine also resulted in a fluorescence decrease similar in magnitude to that observed for glutamate, consistent with the results obtained for the transport currents observed in EAAT2 expressing oocytes (Figure 2B). These results confirm that in contrast to its structural analog cysteine, selenocysteine is an effective substrate for EAAT2.

In HEK293 cells co-expressing mEGFPpH and EAAT3, perfusion with 1 mM selenocysteine or 1 mM glutamate induced a fluorescence decrease, consistent with selenocysteine being a substrate for EAAT3. In contrast to the pH decrease observed for both selenocysteine and glutamate, perfusion of 1 mM cysteine in EAAT3 expressing cells resulted in an increase in mEGFPpH fluorescence, indicating a pH_i_ increase (Figure 6B). The absence of such a fluorescence increase in EAAT2 expressing cells (Figure 6A) or in untransfected control cells (data not shown), suggests that the observed intracellular alkalinization directly correlates with cysteine transport through EAAT3. This result was unexpected as previous reports had indicated that cysteine transport resulted in no change in pH_i_ [7].

The bar graphs in Figure 6A and 6B quantify and compare the slopes of the fluorescence changes induced by selenocysteine and cysteine normalized to the slope of the response induced by 100 µM glutamate. For EAAT2 and EAAT3, 100 µM selenocysteine produced fluorescence changes of 0.85±0.02 (n = 4) and 0.86±0.03 (n = 6) respectively compared to that produced by 100 µM glutamate. In EAAT2-expressing cells, cysteine perfusion produced no observable fluorescence changes, however in EAAT3-expressing cells the normalized slope of the fluorescence change was 0.28±0.03 (n = 6) but opposite to that of selenocysteine or glutamate. Thus, under the conditions used, which favor inward transport of all three substrates, the impact on intracellular proton concentration due to cysteine transport by EAAT3 is distinct from that of the acidic substrates glutamate and selenocysteine.

To provide additional support that this fluorescence increase was due to EAAT3 transport activity, we examined the effect of the EAAT-selective inhibitor TBOA on the cysteine-induced fluorescence increase. In HEK293 cells expressing EAAT3 and mEGFPpH, the increased fluorescence induced by perfusion of 1 mM cysteine was subsequently blocked by co-application of 100 µM TBOA, with fluorescence subsequently returning to baseline (Figure 6C, left). This return to baseline during cysteine perfusion was not observed in the absence of TBOA (Figure 6C, right). These results indicate that in contrast to the acidification observed with EAAT3-mediated transport of selenocysteine or glutamate, the process of cysteine transport results in cytoplasmic alkalinization.

### Release of intracellular substrate pools resulting from EAAT transport activity

As EAATs have been shown to release intracellular substrates due to the reversibility of steps in the transport process [13], [15], [37], pH_i_ increases associated with cysteine transport by EAAT3 could be explained by transporter reversal, which would result in release of internal substrates and the outward co-transport of sodium and protons. To test this possibility, we performed assays monitoring the release of intracellular [^3^H]-L-glutamate and [^35^S]-L-cysteine induced by extracellularly-applied glutamate or cysteine in cells expressing EAAT3. These experiments were performed in an oocyte expression system because they have a low background of endogenous cysteine transport. For these experiments, we also co-expressed ASCT1, an obligate exchanger, to provide a positive confirmation that intracellular cysteine pools are sufficiently large to allow cysteine release by exchange or reverse transport.

**Figure 7:**
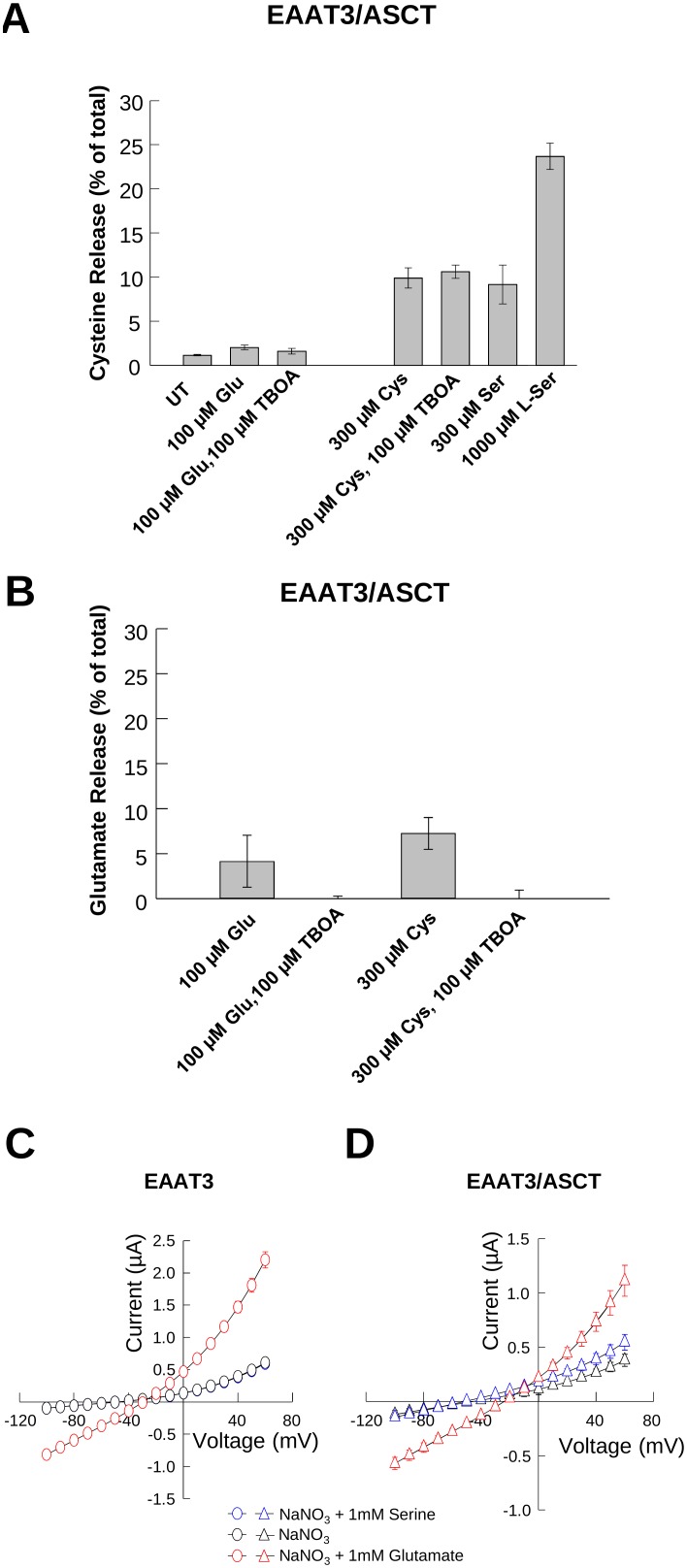
EAAT3 dependent release of [^3^H]-L-glutamate or [^35^S]-L-cysteine. **A and B)** Release of [^35^S]-L-Cysteine (**A**) or [^3^H]-L-Glutamate (**B**) from oocytes co-expressing EAAT3 and ASCT, in response to different buffers and conditions **C)** Averaged current-voltage relationships recorded from oocytes expressing EAAT3 alone (n = 6) or co-expressed with ASCT1 (n = 4) in response to a family of voltage pulses in the absence (black symbols) and the presence of 1 mM serine (blue symbols) or 1 mM glutamate (red symbols). The solid line represents un-injected oocytes in the presence of 1 mM glutamate and 1 mM serine (n = 3).

When oocytes co-expressing EAAT3/ASCT1 were preloaded with [^35^S]-L-cysteine and incubated with 100 µM glutamate, less than 2% of the radiolabel could be detected in the extracellular medium. This release was not significantly inhibited by the transport inhibitor TBOA, and was not substantially greater than that observed under control conditions of buffer alone (Figure 7A). One explanation for this low level of apparent [^35^S]-L-cysteine reverse transport by EAAT3 could be that free cytoplasmic [^35^S]-L-cysteine is rapidly reduced by incorporation into molecules such as glutathione or other metabolic pathways and therefore unavailable for release. To test this, we looked at the effect of transport by the obligate exchanger ASCT1 on the release of internal [^35^S]-L-cysteine. Incubation of the oocytes with 300 µM cysteine, a substrate for both EAAT3 and ASCT1, resulted in the release of 10% of the internal [^35^S]-cysteine, a 5-fold increase over that released by glutamate application. This release was not inhibited by TBOA, consistent with release through ASCT1 and not through EAAT3. L-serine, an ASCT1 substrate with very low affinity for transport by EAAT3 [3], [17], [38], [39], also induced release of [^35^S]-L-cysteine. Incubation of the oocytes in buffer-containing 300 µM and 1 mM L-serine induced release of 10% and 20% of the [^35^S]-L-cysteine respectively, demonstrating that a portion of the [^35^S]-L-cysteine remains unincorporated and available for release by ASCT1, but is not readily released by EAAT3. This would indicate that the low level of cysteine release by EAAT3 is not due to low intracellular substrate availability, but rather the inability of EAAT3 to bind or translocate intracellular cysteine stores.

In contrast, when cells were loaded with [^3^H]-L-glutamate we observed increased release of the radiolabeled substrate when either glutamate or cysteine was applied compared to buffer alone, indicating that glutamate can be readily released by EAAT3. Incubation of the oocytes in buffer containing 100 µM L-glutamate resulted in the release of 5% of the loaded [^3^H]-L-glutamate which was blocked by co-incubation with TBOA (Figure 7B). Substitution of the Na^+^ containing buffer for K^+^ containing buffer, a condition which favors reverse transport, induced release of 2% of the loaded [^3^H]-L-glutamate, with co-application of TBOA blocking this release (data not shown). Cysteine also induced release of 7.5% of the [^3^H]-L-glutamate, which was also blocked by the transport inhibitor TBOA and was not significantly different from that observed for glutamate (Figure 7B). Incubation with 1 mM L-serine, which is not transported by EAAT3 [3], [17], [38], [39], did not induce significant release of loaded [^3^H]-L-glutamate above control levels (data not shown). Taken together these data suggest that although glutamate can be readily exchanged, cysteine transport by EAAT3 is unidirectional.

EAAT3 and ASCT1 show an uncoupled anion conductance which is activated by Na and enhanced upon application of their respective substrates glutamate and serine [31], [32]. To corroborate ASCT1 co-expression with EAAT3 in these experiments, we measured substrate gated anion currents mediated by EAAT3 and ASCT1, which are gated by glutamate and serine respectively. Using two electrode voltage-clamp recordings, we measured substrate-gated NO_3_ currents mediated by EAAT3 and ASCT1. Figure 7C shows that in oocytes expressing EAAT3 alone, at +60 mV we obtained an average macroscopic current amplitude of 0.61±0.05 µA (black circles, n = 6) after perfusion of NaNO_3_ in the absence of substrates, no additional current was detected upon application of 1 mM serine, with an average current amplitude of 0.60±0.05 µA (blue circles, n = 6) (Figure 7C). Application of 1 mM glutamate, elicited a current 3-fold larger, with average amplitude of 2.2±0.12 µA (red circles, n = 6), as previously observed [33]. In comparison, for oocytes co-expressing EAAT3 and ASCT1 we obtained a macroscopic current amplitude of 0.4±0.05 µA (n = 4) in the absence of substrate, which was enhanced upon application of both serine (0.6±0.07 µA, blue triangles, n = 4) and glutamate (1.1±0.1 µA, red triangles, n = 4) (Figure 7D). Current amplitudes from control oocytes in the presence of substrate were 0.1±0.007 µA (solid lines, n = 3). This experiment demonstrates that EAAT3 and ASCT1 are indeed co-expressed in these cells.

## Discussion

To provide additional insight into the mechanism of cysteine transport through EAAT3, we compared transport properties of a cysteine structural analog, selenocysteine, with those of the well-established EAAT3 substrates, cysteine and glutamate. By monitoring the transport of substrates and co-transported ions using uptake, transport currents and a pH-biosensor, we suggest a mechanism by which EAAT3 facilitates cysteine binding and transport through cysteine deprotonation. Although similar to cysteine, selenocysteine differs in the protonation and charge of the amino acid side chain at physiological pH. With a pK_a_ = 5.3 (cysteine pK_a_ = 8.3) selenocysteine is deprotonated and negatively charged at physiological pH, whereas cysteine is primarily protonated and neutral. The ability of EAATs to selectively transport negatively charged substrates such as glutamate or aspartate has been proposed to be largely conferred by an arginine residue (R447 in EAAT3) that is uniquely positioned in the binding site to interact with the negative charge of substrate side chains. Replacement of this residue by cysteine alters the EAAT3 substrate specificity to neutral amino acids such as serine, alanine and cysteine [17]. Transport of the negatively charged selenocysteine would be facilitated by interaction with the R447 in the binding site in a manner similar to glutamate and other acidic substrates. This could explain the higher affinity of selenocysteine for transport and lower IC_50_ for inhibition of glutamate transport compared to cysteine (Figure 3). Transport would proceed as with other acidic substrates, with the negatively charged selenocysteine binding extracellularly along with a co-transported proton and sodium ions, followed by translocation and intracellular release. With selenocysteine remaining deprotonated in the neutral pH of the cytoplasm, the release of the co-transported proton would result in a net pH_i_ decrease, similar to that observed for glutamate (Figure 6A, B).

Although selenocysteine and cysteine have structural and chemical similarities, a primary difference between these molecules is the protonation state at physiological pH. With a higher pK_a_ for cysteine (pK_a_ = 8.3), at pH = 7.5 ∼14% of the cysteine molecules would approach the binding site as the negatively charged thiolate, which could then bind to the transporter in similar fashion as do glutamate and selenocysteine. The remaining uncharged thiol form of cysteine would bind in a manner more similar to serine, which is a poor EAAT substrate with low affinity for the transporters [3], [17], [38], [39]. However given the appropriate conditions, cysteine could be induced to deprotonate and form the negatively charged thiolate. Transport of cysteine by EAAT3 may be facilitated due to a micro-environment at the binding site which facilitates cysteine deprotonation, with neutral cysteine approaching the binding site, deprotonating and binding as would glutamate or selenocysteine. The low cysteine transport activity displayed by other EAATs (Figure 2) may reflect a difference in the binding site environment that does not readily facilitate cysteine deprotonation.

Transport of a deprotonated cysteine is likely to be accomplished by the same transport mechanism as glutamate and other acidic EAAT substrates as previously proposed [11]. Deprotonated cysteine bound in the substrate-binding site along with the co-transported Na^+^ and proton would be translocated and released intracellularly. Upon release from the binding site the cysteine thiolate would readily re-protonate in the cytoplasm largely neutralizing the co-transported proton. The lack of intracellular acidification during cysteine transport observed here and previously [7] is consistent with such a mechanism [11]. However, to explain the pH_i_ increase we observe with cysteine uptake by EAAT3 we also need to consider substrate exchange and the reversibility of transport [15], [16]. The inward translocation of the cysteine thiolate and co-transported Na^+^ and H^+^ is accompanied by a lower rate of outward translocation of intracellular substrates and co-transported ions. If cysteine were exchanged with cysteine (homo-exchange), the process would be pH neutral. However, if the exchanging substrate were glutamate or aspartate (hetero-exchange) which are in mM concentrations within cells [7], the largely pH-neutral inward translocation of cysteine would be accompanied by glutamate efflux together with a proton. The cytoplasmic alkalinization observed with cysteine transport (Figure 6) together with the inability of EAAT3 to release intracellular cysteine (Figure 7A) are consistent with the observed pH_i_ increase resulting from cysteine-glutamate heteroexchange. Detection of this pH_i_ increase using mEGFPpH, which was not seen using BCECF [7], may have been due to use of the membrane attached mEGFPpH positioned proximal to the site of ion flux which may provide additional sensitivity compared to the cytoplasmically-distributed BCECF. These results highlight the advantages of using a membrane localized biosensor for monitoring transported mediated ionic fluxes in real time [27].

The mechanism of cysteine deprotonation prior to transport suggests that the thiol form of cysteine would require an additional step prior to translocation whereas the thiolate form would bind as do the other acidic substrates. This is supported by our observations of the effect of pH on glutamate inhibition of cysteine transport (Figure 4). We observed that 300 µM cysteine transport at pH 6.9 (4% thiolate) was more effectively inhibited by glutamate than at pH 8.5 (61% thiolate). The thiolate would readily interact with the residues of the substrate-binding site, particularly R447, while the cysteine thiol would require additional steps to form the proper interactions and may be readily displaced by acidic substrates such as glutamate. This would also provide an explanation for the very low affinity of serine for the transporters, as serine is fully protonated at neutral pH. However, when R447 is replaced by an uncharged cysteine residue, serine and alanine are readily transported by EAAT3/EAAC1 [17].

Our results also demonstrate that selenocysteine is transported by the plasma membrane excitatory amino acid transporters (EAATs). This is the first evidence identifying specific plasma membrane transporters of the essential amino acid selenocysteine. Characterization of the transport properties of selenocysteine show that it is a high affinity substrate for the human isoforms EAATs 1–3, with properties similar to other acidic EAAT substrates and in contrast to its structural analog cysteine, which is efficiently and selectively transported through EAAT3. Selenocysteine forms the active site in a number of cellular proteins such as glutathione peroxidase, a critical peroxide scavenging enzyme. Free cytoplasmic selenocysteine is not specifically incorporated into the active site of this and other enzymes but can be randomly incorporated into cysteine sites as a result of non-specific incorporation into the Cys tRNA (tRNA^Cys^) (for review, see [40]). Elemental selenium derived from selenocysteine, can be incorporated through a selenophosphate intermediate into special selenocysteine tRNAs (tRNAs^Sec^) which are used during translation of selenoproteins [23]. Transport of selenocysteine into the cell by EAATs may play an important role to facilitate the production of tRNA^Sec^ by working to maintain the intracellular pool of selenocysteine for this process.

Cysteine transport by EAAT3 has been proposed to be critical *in vivo* for maintaining a sufficient intracellular pool of free cysteine for glutathione synthesis [4]. The expression of EAAT3 in neurons and the unique cysteine transport properties compared to the reduced cysteine transport by the glial expressed EAATs support this idea. As we observed, EAAT3 readily transports cysteine into cells with little detectable release due to EAAT3 transport activity. The absence of significant cysteine release by EAAT3 is likely the result of a much lower substrate affinity at the intracellular binding site [11], [12], low intracellular cysteine concentrations (<200 µM) maintained by cells [4], or local environment surrounding the intracellular binding site that is not conducive to thiolate formation. Also, other substrates such as glutamate are present at concentrations above 1 mM [7] and thus more likely to participate in the exchange process. This is in contrast to ASCT1, which transports neutral amino acids in an obligate exchange mode and which we observed to facilitate the release of intracellular cysteine in the presence of extracellular serine (Figure 7). In neurons, loss of cysteine due to ASCT1 transport activity could be mitigated by reuptake through EAAT3. Release of cysteine by glial-localized ASCT1 could provide a mechanism to facilitate the transfer of cysteine into neurons. In both cases it would be advantageous for the glial carriers, EAAT1 and EAAT2, to transport cysteine poorly thus increasing the probability of capture by neuronal EAAT3.
